# Surprisal analysis of genome-wide transcript profiling identifies differentially expressed genes and pathways associated with four growth conditions in the microalga *Chlamydomonas*

**DOI:** 10.1371/journal.pone.0195142

**Published:** 2018-04-17

**Authors:** Kenny A. Bogaert, Sheeba S. Manoharan-Basil, Emilie Perez, Raphael D. Levine, Francoise Remacle, Claire Remacle

**Affiliations:** 1 Theoretical Physical Chemistry, UR MOLSYS, University of Liège, Liège, Belgium; 2 Genetics and Physiology of Microalgae, UR InBios, University of Liège, Liège, Belgium; 3 The Fritz Haber Research Center for Molecular Dynamics, Institute of Chemistry, Hebrew University of Jerusalem, Jerusalem, Israel; 4 Department of Molecular and Medical Pharmacology, David Geffen School of Medicine, University of California Los Angeles, Los Angeles, California, United States of America; Universidade de Lisboa Instituto Superior de Agronomia, PORTUGAL

## Abstract

The usual cultivation mode of the green microalga *Chlamydomonas* is liquid medium and light. However, the microalga can also be grown on agar plates and in darkness. Our aim is to analyze and compare gene expression of cells cultivated in these different conditions. For that purpose, RNA-seq data are obtained from *Chlamydomonas* samples of two different labs grown in four environmental conditions (agar@light, agar@dark, liquid@light, liquid@dark). The RNA seq data are analyzed by surprisal analysis, which allows the simultaneous meta-analysis of all the samples. First we identify a balance state, which defines a state where the expression levels are similar in all the samples irrespectively of their growth conditions, or lab origin. In addition our analysis identifies additional constraints needed to quantify the deviation with respect to the balance state. The first constraint differentiates the agar samples versus the liquid ones; the second constraint the dark samples versus the light ones. The two constraints are almost of equal importance. Pathways involved in stress responses are found in the agar phenotype while the liquid phenotype comprises ATP and NADH production pathways. Remodeling of membrane is suggested in the dark phenotype while photosynthetic pathways characterize the light phenotype. The same trends are also present when performing purely statistical analysis such as K-means clustering and differentially expressed genes.

## Introduction

*Chlamydomonas reinhardtii* is a unicellular green microalga which has been a reference organism for photosynthetic studies for decades [1]. With the completion of the sequencing of its nuclear genome [2], *Chlamydomonas* has also become a model of choice for expression studies aimed at dissecting acclimation to various conditions and perturbations (excess light, variations of CO_2_ concentrations, nutrient deprivation, metal stress, etc.) using -omics strategies [3]. Until now, most of the -omics analyses have been performed when cells are cultivated in the light and liquid medium. However, the natural habitat of *Chlamydomonas* spp. also includes distinct environmental niches such as soil (the source of the *C*. *reinhardtii* strain), glacier (*C*. *nivalis* also known as snow alga) and ponds [1], which means that cells may also encounter periods of darkness and assimilate organic compounds.

Cultivations in the light or in darkness, but also in liquid or on solid medium are thus representative of what *Chlamydomonas* may experience in its natural environment. In addition, looking at algal expression in non-standard conditions is also justified as immobilized microalgae on solid-state photobioreactors represent a growing field of investigation for production of high value compounds [4] and wastewater remediation [5]. Moreover growth in fermenters may lead to higher biomass and lipid yields than in the light [6]. To understand the main characteristics of cell expression in the four different conditions mentioned above (agar@light, agar@dark, liquid@light, liquid@dark), we performed a transcriptomics analysis. The RNA-seq response data coming from samples of two different labs grown in the four growth conditions were examined using surprisal analysis. Surprisal analysis is a thermodynamic approach which provides a biophysicochemical understanding and quantitative characterization of -omics data using a molecule centered approach. It has been applied successfully for transcriptomics expression levels in human cells [7–11] and recently on metabolic data in *C*. *reinhardtii* [12]. The method allows defining a balance state, also called steady state, common to all the types of samples. In the balance state, the transcript levels for all the growth conditions are identical within experimental error. Therefore, the balance state can serve as a reference to which the measured transcript levels can be compared. In surprisal analysis, the deviations of the transcript levels with respect to the balance state are quantified by constraints that characterize their response to a perturbation or variables influencing the transcriptome [7–11]. We concluded that the first constraint differentiates between agar-grown and liquid-grown phenotypes, while the second constraint differentiates the dark-grown and light-grown ones. First and second refer to the importance of the two constraints as determined by the analysis. In the present case however the second constraint is almost as important as the first. Gene families contributing the most to the first and second constraint are identified.

We compared the results of surprisal analysis to conventional purely statistical methods currently used to analysis gene expression levels: K-means clustering and differentially expressed genes. The main difference is that the purely statistical analyses are carried out on mean centered data, while surprisal analysis yields a balance state compared to which the changes due to the different growth conditions are quantified on a thermodynamical basis [7,8,10,13] (see Material and methods below). The balance state represents a stable steady state of minimum free energy. In the balance state, each gene has a prior thermodynamic weight and those are not uniform. The constraints provide a measure on how much the free energy of a gene in given ‘growth condition’ sample deviates from its thermodynamic weight in balance state due to the unbalanced processes that correspond to the phenotype of constraint. Despite the fact that surprisal analysis and purely statistical analysis use measures of a different nature, both type of analysis yield to similar phenotypic trends.

## Material and methods

### Strains and growth cultivation

The wild-type reference strain of our laboratory derived from the 137C strain [14] was used for the analysis of samples grown on agar plates. For that purpose, the strain was serially diluted and isolated colonies were cultured on agar plates at 25°C, in low light (50 μE.m^-2^.s^-1^) and acetate (17 mM, Tris-Acetate-Phosphate, TAP medium) [15], in darkness and TAP, or in darkness TAP + peptone (0.1%) to boost growth in the dark. Colonies were picked up for RNA-seq analysis when they reached 0.5–0.8 cm of diameter, corresponding to 5x10^5^ to 1x10^6^ cells per colony, which represents 10 days of cultivation for light-grown colonies and 3 weeks for dark-grown colonies. For liquid grown samples, a complemented version of our reference strain was used, the *iclC* strain. The *iclC* strain is very similar to our reference strain as described in [12,16]. *iclC* was inoculated from a 48 h liquid preculture into a sterilized Multi-Cultivator MC 1000-OD (Photon Systems Instruments) containing 80 mL of Tris-Phosphate [15], buffered with HCl at pH 7.0 with specific acetate concentrations (17 mM, 31 mM, 44 mM or 57.5 mM, sodium acetate). The experimental cultures were grown under moderate light (50 μE.m^-2^.s^-1^). Two time points of the growth curves (12h and 28h of growth) were chosen for RNA extraction, corresponding to early (≈1x10^6^ cells/mL) and mid-exponential (≈4x10^6^ cells/mL) growth phase. The growth curves were made in triplicate.

### RNA extraction

For agar-grown samples, colonies were frozen at -80°C before RNA extraction. RNA was isolated from individual colonies using RNeasy Qiagen plant kit. For liquid-grown samples, 1.5x10^7^ cells were pelleted for RNA extraction at time point 12h and 5.5x10^7^ cells at time point 28h. RNA was extracted according to [17]. RNA samples were quantified by Ribogreen and those passing the quality control (Bioanalyzer, Agilent technologies, Agilent 2100 Expert software) were selected for cDNA synthesis.

### Sequencing

Library preparation started with 100 ng total RNA for agar-grown samples 500 ng total RNA for liquid-grown samples. Illumina Sequencing (SE 1x75 on a NextSeq500 machine) was performed at the GIGA-R Sequencing platform (University of Liège) following manufacturer’s protocol (Illumina Inc, San Diego CA, USA).

### Read trimming and quality filtering

Read quality was assessed with FastQC v.0.11.5 (www.bioinformatics.babraham.ac.uk/projects/). No significant problems were observed.

Quality filtering of RNA-seq samples was done on single-end reads using trimmomatic (v0.36) [18], removing low quality sequences (average Q20 over a 4-base sliding window, minimum length = 50 bp with a leading and trailing quality threshold of Q25).

### Read mapping

Mapping of the reads to the *Chlamydomonas reinhardtii* genome v5.5 assembly [2] was done using STAR [19] with default presets except for intron size (-alignIntronMin 20 and -alignIntronMax 3000). More than 12 million uniquely mapping reads were mapped per sample (S1–S4 Tables). Agar-grown sample 18_2 showed a particular low yield of reads and a low fraction of uniquely mapping reads (19%). Therefore this sample was omitted from the data set (S1 Table). The uniquely mapping reads were assigned to the primary transcripts using cuffquant and cuffdiff (v2.2.1) with the default fragment size of 200 and standard deviation of 80 [20]. Expression estimates were normalized to library size and gene length by cufflinks to calculate the FPKM values (S5 Table).

### Surprisal analysis

Surprisal analysis is based on thermodynamical entropy [7,8,10,13,21] and therefore is carried on the logarithm of the gene expression levels.

In editing the data for surprisal analysis all transcripts with an average FPKM value lower than 1 based from the agar grown colonies were removed because most of the noise is due to low expression values, in particular those below 1 FPKM [22]. In total 12774 genes were kept in the data set. Values lower than 0.01 FPKM were substituted with 0.01 FPKM to allow the computation of logarithms and expression ratios (S5 Table).

The natural logarithm (*Y*_*i*_(*s*), where *i* stands for a gene and s for a sample) of the *N* = 12774 gene expression values, *X*_*i*_(*s*), in each of the 38 samples was subjected to surprisal analysis [7–11], (tutorial in [21]). The values *Y*_*i*_(*s*) are arranged in a *N* x *N*_*s*_ rectangular matrix **Y**, where *N*_*s*_ = 38 is the number of samples. The constraints, *G*_*iα*_, and Lagrange multipliers, λ_α_(*s*) are determined via the singular value decomposition (SVD) of **Y** as described by [8].

$$Y_{i}\left(s\right)=\text{ln}X_{i}\left(s\right)=\text{ln}X_{i}^{0}+\sum_{\alpha =1}^{N_{s}}G_{i\alpha }\lambda_{\alpha }\left(s\right)$$ (1)

Here *α* is the index of constraints, *N*_*s*_ is the total number of samples, *i* is the index of the gene and *s* is the index of the sample. The expression for *G*_*iα*_ and *λ*_*α*_(*s*) are given by the eigenvectors and the eigenvalues of the SVD of the matrix **Y**:

$$G_{i\alpha }=U_{i\alpha }\;\text{and}\;\lambda_{\alpha }\left(s\right)=\omega_{\alpha }V_{\alpha s}$$ (2)

where **U** and **V** are respectively the left and right eigenvectors of the **Y** matrix as determined by the SVD procedure and ω_*α*_ the singular values. The eigenvalues of the **Y** matrix are ordered by decreasing order and when all the *N*_*s*_ terms are kept, the surprisal expression of the transcript levels given in Eq (1) is an *exact* representation of the data. Usually just a few terms in Eq (1) (smaller than the number of samples *N*_*s*_) suffice to describe the input. Each constraint *α* corresponds to a given phenotype. For a given value of *α*, the surprisal analysis allows for a factorization between the weight of the constraint, *G*_*iα*_, on a given gene *i* and the Lagrange multiplier, *λ*_*α*_(*s*), that is the weight of sample *s* in the phenotype that corresponds to the constraint *α*.

In the first term of Eq (1), $\text{ln}X_{i}^{0}=G_{i0}\lambda_{0}$, corresponds to the prior thermodynamical weight of the gene ‘*i’* in the balanced state. The balance state is this stable state that is common to all the colonies and with respect to which the changes in the gene expression levels due to the successive constraints, *α* = 1, …, *N*_*s*_, are expressed. The larger is the prior thermodynamical weight of a gene *i*, *G*_*i*0_*λ*_0_, the more stable it is, and the lower is its free energy which is given by $-\text{ln}X_{i}^{0}=-G_{i0}\lambda_{0}$. The constraints provide a quantitative measure of the deviation with respect to the balance state. By plotting the values of the Lagrange multipliers for the different colonies for a given constraint *α*, one can identify different groups of samples that differ by the sign of their Lagrange multiplier, *λ*_*α*_(*s*) for the phenotype *α*. In particular, we show above that for the first constraint, *α* = 1, samples grown on agar and those grown in liquid have an opposite sign of their Lagrange multipliers. The analysis of the weights of the corresponding phenotype vector, *G*_*iα*_, over the genes in terms of pathways gives access to the different pathway contributions to the phenotype agar-grown versus liquid-grown. For *α* = 2, samples grown in the dark and samples grown in the light are characterized by Lagrange multipliers of different signs. The analysis of the corresponding phenotype allows identifying the pathways that contribute most to the growth in dark and light conditions respectively.

### Differential gene expression in the constraint vector *G*_*iα*_

Genes of the phenotype associated with each constraint *α* were ranked according the value of the weight *G*_*iα*_. According to this ranking, 100 smallest and largest values were considered differentially expressed for each phenotype. In the case of the balance state, genes that correspond to a term *G*_*i*0_*λ*_0_ > 0 are the most stable and those for which *G*_*i*0_*λ*_0_ > 0 are unstable. The latter are the genes that will appear with the largest and the smaller weights in the phenotypes associated with the constraints and therefore will be the most differentially expressed in the constraints, *α* = 1, …, *N*_s_.

### Gene set enrichment

In [7], differential expression of gene ontology classes have been assessed using hypergeometric tests on differentially expressed genes for the different constraints. Here we developed a complementary approach, which consists of assigning a weight to each pathway in a given phenotype described by the constraint *α*. This approach has the advantage to take into account the weights of all the genes, *G*_*iα*_, in a given constraint and therefore do not to depend on the number of genes (typically 100) kept in the differential gene expression analysis. The two approaches are complementary because pathways that comprise several genes that have a high weight in a given phenotype (and therefore appear in the genes most differentially expressed) will have a large weight.

Genes were categorized in gene sets using the Kyoto Encyclopedia of Genes and Genomes (KEGG) (http://www.genome.jp/kegg/) and the functional annotation info for *C*. *reinhardtii* v5.5 predicted proteins were obtained from the correspondence table downloaded from Phytozome.

Pathways that correspond to a gene set with less than 10 genes were omitted from the dataset. For each constraint, *α*, of interest, each subset of genes was divided in two subsets according to the sign of their weight *G*_*iα*_. For a given set of genes that corresponds to the pathway *J*, the *G*_*iα*_ values for genes that are respectively larger or smaller than zero were summed together to get respectively the positive (P) and negative (N) weight of the pathway for constraint *α*:

$$P_{\alpha }^{J}=\sum_{i=1}^{N_{j}}G_{i\alpha }^{2}\;\text{for}\;G_{i\alpha }\;>\;0$$ (3)

$$N_{\alpha }^{J}=\sum_{i=1}^{N_{j}}G_{i\alpha }^{2}\;\text{for}\;G_{i\alpha }\;<\;0$$ (4)

The ratio

$$SR_{J}=P_{\alpha }^{J}/N_{\alpha }^{J}$$ (5)

is a measure for the contribution of the gene set of pathway *J* to constraint α. In Eq (5), *N*_*J*_ is the number of genes in pathway *J*.

Set ratios, *SR*_*J*_, were ordered according their value describing their importance for the described phenotype. These gene sets where all values *G*_*iα*_ are either positive or negative, were subsequently ranked on $P_{\alpha }^{J}$ or $N_{\alpha }^{J}$ respectively.

**Fig 1:**
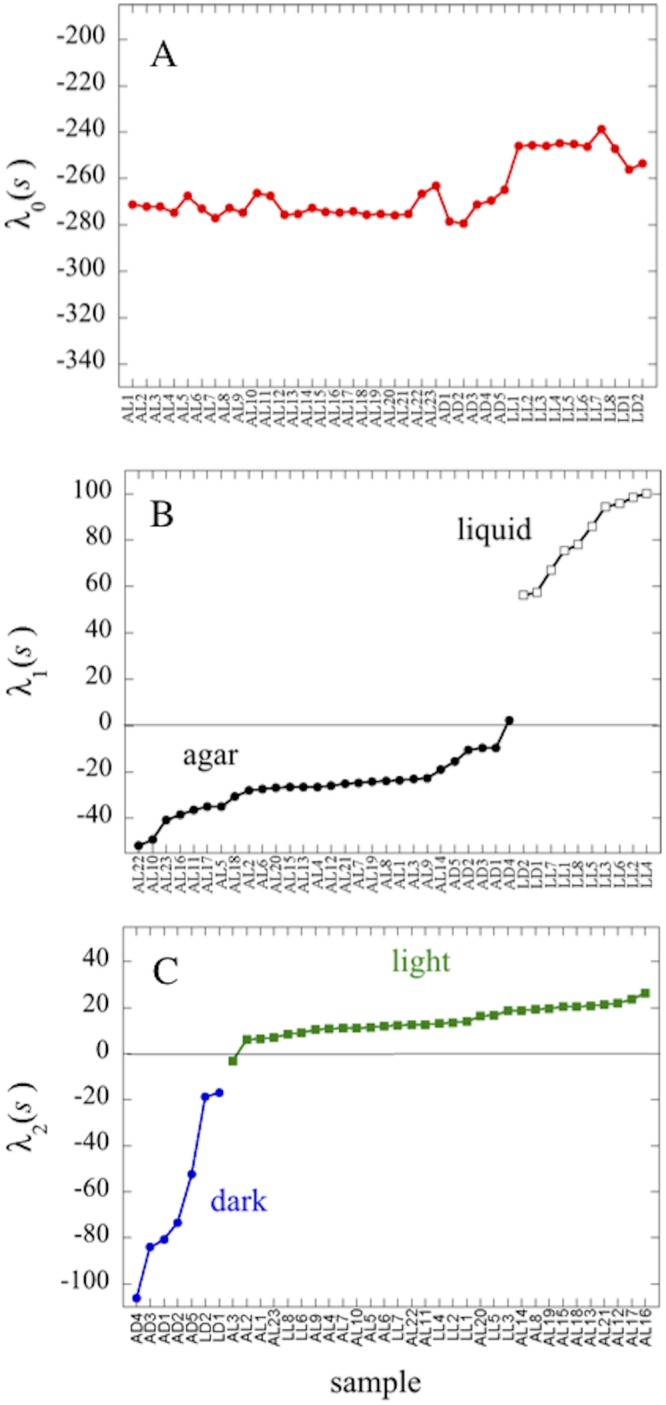
Lagrange multipliers values for the balance state (λ_0_(*s*)), the first (λ_1_(*s*)) and the second (λ_2_(*s*)) constraint. (A) λ_0_, (B) λ_1_ and (C) λ_2_ values are determined using the 38 samples, see Methods.

**Fig 2:**
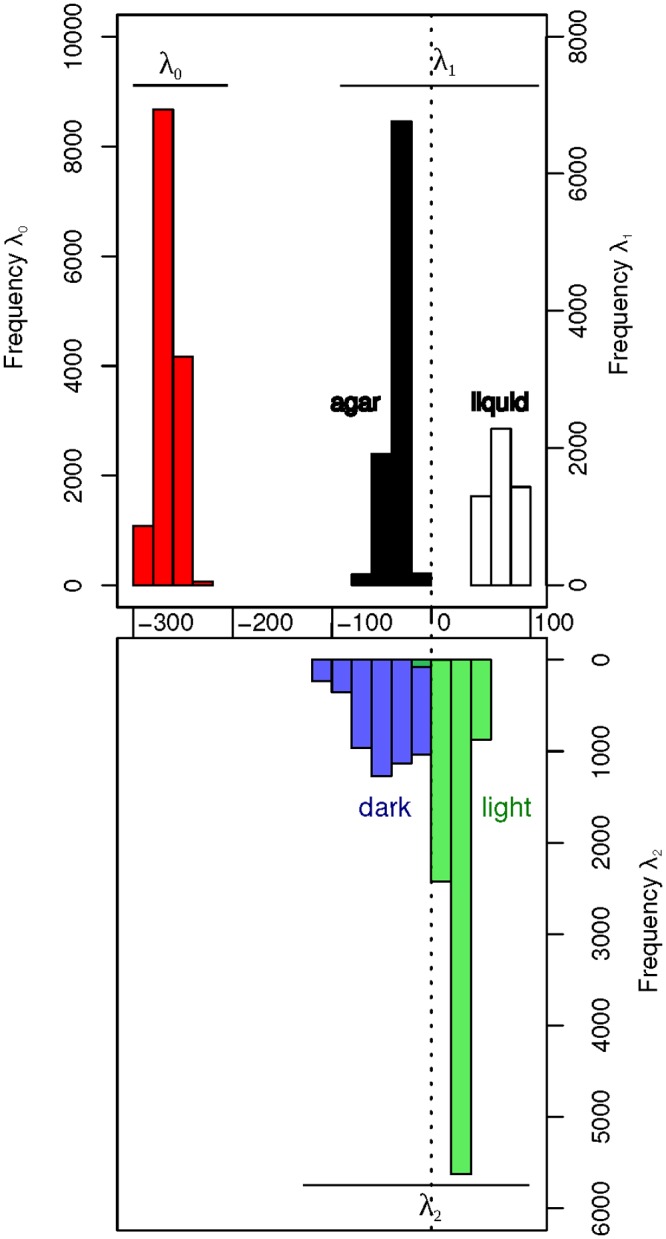
Lagrange multipliers values for the balance state (λ_0_(*s*)), the first (λ_1_(*s*)) and the second (λ_2_(*s*)) constraint. The λ_0_(*s*), λ_1_(*s*) and λ_2_(*s*) values are determined using 1000 random combinations of 14 samples out of the 38 available.

Both low ratios and high ratios are predicted by surprisal analysis to be important for the phenotype and to be enriched in their respective phenotypes. For the balance state, genes that correspond to a term *G*_*i*0_*λ*_0_ > 0 are the most stable and those for which *G*_*i*0_*λ*_0_ < 0 are unstable. For the first phenotype, genes which correspond to a term *G*_*i*1_*λ*_1_(*s*) >0 are overexpressed for samples grown in liquid conditions and underexpressed for samples grown on agar while genes for which *G*_*i*2_*λ*_2_(*s*) >0 are overexpressed in the light conditions and underexpressed in dark ones. Since the values of the Lagrange multiplier, *λ*_1_(*s*), are positive for the colonies grown in liquid phase and negative for those grown on agar (see Figs 1 and 2), high SR pathway ratios correspond to gene sets that are overexpressed for samples grown in liquid conditions and low SR ratios correspond to gene sets that are over expressed for samples grown on agar. For the second constraint, samples grown in light conditions have a positive Lagrange multiplier *λ*_2_(*s*) while samples grown in the dark have negative *λ*_2_(*s*) values. So high SR pathway ratios correspond to gene sets that are over expressed for samples grown in light conditions while low SR ratios values correspond to gene sets that are over expressed in dark grown samples.

### Randomization

1000 random combinations of biological replicates of the AL (6), AD (3), LL (3) series were drawn to confirm the consistency of the surprisal analysis under influence of biological variation. The 2 samples from the LD series (LD1 and LD2) from [23] were always included due to the low number of replicates available in the study. The clustering of λ_1_(*s*) and λ_2_(*s*) into two groups, liquid versus agar and dark versus light for each drawn subset was tested using a Wilcoxon t-test. The threshold for of the pvalue was set to <0.05. In all subsets two groups corresponding to the two variables, λ_1_(*s*) and λ_2_(*s*), could be identified. Because the sign of λ_0_(s) and G_iα_ is a convention selected automatically by the SVD procedure, they differ between subsets. Signs were reattributed to a chosen convention depending on the sign of G_iα_ of one of the most enriched genes for the experimental variable in the analysis of all samples.

### DGE analysis

Expression levels (FPKM) were square root transformed and tested for differential expression using CLC Genomics Workbench (10.0.1) by ‘Exact Test’ for two-group comparisons [24] using a total count filter cutoff of 5.0 and gene specific estimation of tag-wise dispersions. Genes that had a fold change of > 2 and FDR-corrected P value of < 0.5 were judged to be significantly differentially expressed.

### K-means clustering

The KMC K-means algorithm of MeV (version 4.8.1) [25] was implemented to cluster the 12774 genes. FPKM values were ln-transformed and mean-centered at the gene-level. The figure of merit algorithm was used to estimate the appropriate number of clusters. K-means clustering using Pearson’s correlation as a measure was then applied to separate the genes into 4 groups of coregulated genes.

## Results and discussion

To obtain transcriptomics data of samples cultivated on agar plates, serial dilutions of our reference wild-type strain derived of the 137C strain [14] were performed to get between 15 and 80 isolated colonies per plate and plates were transferred in low light (50 μE.m^-2^.s^-1^) or in the dark, on acetate containing medium (17 mM). Individual colonies were picked up for RNA-seq analysis when they reached 0.5–0.8 cm of diameter, corresponding to 5x10^5^ to 1x10^6^ cells per colony (S1 Fig). RNA seq data were obtained from 23 colonies grown on agar in the light, named AL for Agar-Light (AL1-AL23) and 5 colonies grown on agar in the dark, named AD for Agar-Dark (AD1-AD5) (S1 and S2 Tables). Transcriptomics data of cells cultivated in liquid medium and in the light (50 μE.m^-2^.s^-1^) were obtained using a similar strain [12,16] grown at different acetate concentrations (17 mM, 31 mM, 44 mM and 57.5 mM). Samples were harvested at two time points of the growth curve corresponding to the early (1x10^6^ cells/mL) and the mid-exponential (4x10^6^ cells/mL) phase, named LL for Liquid-Light (LL1-LL8) (S3 Table). Transcriptomics data of cells cultivated in the dark in liquid medium in the presence of acetate (17 mM) were obtained from [23] using another reference strain also derived from the 137C strain [23] and named LD for Liquid-Dark (LD1-LD2) (S4 Table). Surprisal analysis of the RNA seq data from the 38 samples (AL1-AL23, AD1-AD5, LL1-LL8, LD1-LD2) was then carried out in order to characterize gene expression. Surprisal analysis is a methodology that identifies constraint(s) explaining the phenotype of individual entities which can be single cell lines [7,8,11], tissues in human patients [9] or in our case microalgal cells [12]. Our aim here is to identify the constraints that would allow differentiating and characterizing the different samples.

### The first and second constraints allow discriminating between agar/liquid and dark/light samples respectively

The values of the Lagrange multipliers and of the constraints were computed as described in the ‘Surprisal analysis’ section of Methods. The balance or steady state, that is the reference stable distribution of expression levels common to all samples in the absence of any biological constraint, is defined by the Lagrange multiplier λ_0_(*s*) and the balance state phenotype G_0_. The λ_0_(*s*) values for each sample, *s*, are plotted in Fig 1A. As required for the definition of the balance state, in which the expression levels of the transcripts are expected to be identical for all the samples, the values of λ_0_(*s*) (where *s* stands for sample and the value is the importance of the balanced state) are constant within a range (41 units) that reflects small variations from sample to sample. Thus we first comment that all the 38 samples exhibit a common balance state despite the fact they are obtained from two different laboratories (AL1-AL23; AD1-AD5; LL1-LL8 versus LD1-LD2).

On the other hand, the values of the Lagrange multiplier of the first constraint, λ_1_(*s*) (Fig 1B), have different signs depending on whether the samples were grown on agar or in liquid. λ_1_(*s*) is negative for 27 samples (AL1-AL23, AD1-AD2, AD3, AD5) grown on agar on the 28 analyzed and positive for all the 10 samples grown in liquid (LL1-LL8, LD1-LD2). This result identifies the first constraint as the one that allows discriminating between the agar and liquid samples. The value of λ_1_(*s*) for the sample AD4 is slightly positive and very close to zero. This indicates that the weight of the first constraint is close to zero for this sample grown on agar in the dark, which may reflect slightly different growth conditions compared to the other agar grown colonies. Moreover, the difference between the range of positive and negative values of λ_1_(*s*) is 152 units (Fig 1B), much larger that the range of values of λ_0_(*s*), which confirms that the first constraint is significant for explaining the differences in expression levels with respect to the balance state. The second constraint (Fig 1C) allows the separation between light and dark samples since the seven dark samples (AD1-AD5, LD1-LD2) have negative values of λ_2_(*s*) and 30 samples grown in the light (AL1-AL2, AL4-AL23, LL1-LL8) have positive values. Only one sample grown in the light (AL3) has a slightly negative value, which does not contradict our conclusions for the same reasons as above. The difference between the range of the negative and positive values of λ_2_(*s*) are also much larger that the range of values of λ_0_(*s*) (133 units). The agar versus liquid samples and dark versus light phenotypes segregate from each other with different signs only in the plots of the Lagrange multipliers of the first and the second constraint respectively, indicating that the phenotypes describing agar/liquid and light/dark conditions are completely encapsulated by the contribution of the first and the second constraint to the gene expression levels, see Methods (S6 Table). The pathway analysis made in point 3.2 (see below) also confirms that these two constraints explain these types of growth conditions.

Different strains are used in this study (137C) and the study of [23] (4A+). 4A+ has been derived from 137C and selected for rapid growth on acetate in the dark [26]. Therefore strain specific phenotypes could be characterized by surprisal analysis and associated with a specific constraint. Interestingly, the λ_3_(s) values of the 4A+ samples are separated by a gap of about 60 units from the multipliers of those of the 137C samples, see S2 Fig. We note however that while significantly different in value, the two samples of the 4A+ strain have the same sign of *λ*_3_(*s*) as several of the AL and AD 137 C samples which suggests that the third constraint does not lead to a fully unambiguous strain phenotype characterization. We therefore will not analyze this constraint further.

Results are similar when the surprisal analysis is performed on 1000 random combinations of 14 samples (6 samples from the AL series, 3 samples from the AD series, 3 samples from the LL series, and the 2 samples from the LD series) (Fig 2). λ_0_(*s*) values are equivalent to those found when all the samples are analyzed. λ_1_(*s*) has negative values for the agar-grown samples and positive values for the liquid-grown samples and the values are quite similar to those found when all the samples are analyzed together. In the same way, the values of λ_2_(*s*) are negative for the dark-grown samples and positive for the liquid-grown samples. These results thus demonstrate that the first and the second constraints are robust with respect to sampling and indeed responsible for the difference between agar/liquid and dark/light samples respectively.

### Gene set enrichment analysis allows the description of the biological pathways contributing to the balance state and to the first and second constraints

Surprisal analysis (see Methods) determines a gene transcript expression profile associated with each constraint. This transcript expression profile is given by a vector **G**_α_ where α is the index of the constraint and characterizes the phenotype associated with the constraint. The components G_iα_ of the vector **G**_α_ determine the weight of transcript *i* in the phenotype associated with the constraint α whose Lagrange multiplier is λ_0_(*s*). One can therefore rank the contribution of a transcript to a given phenotype according to its weight, G_iα_. As described in section ‘Gene set enrichment’ of Methods, the annotated genes [2] of *Chlamydomonas* are categorized in gene sets (KEGG: Kyoto Encyclopedia of Genes and Genomes, http://www.kegg.jp/kegg/) using the 1000 random combinations of 14 samples cited above. This categorization therefore allows the identification of gene sets that contribute most to the phenotype associated with a given constraint, α.

From the G_iα_ values computed for each transcript using surprisal analysis, we define a ‘SR ratio’ (see Methods Eqs [3–5]) which quantifies the contribution of each gene set associated with a specific pathway to the phenotype. 113 KEGG pathways are identified in *Chlamydomonas* comprising 3145 genes of which 2992 are found in our analysis (S7 Table). We thus consider that the first 10 pathways are the most representative of a given phenotype. We begin by analyzing the gene set composition of the balance state. Logically, acetate assimilation (Glyoxylate and dicarboxylate metabolism) [27] is found in the balance state, as acetate in the growth medium is the only common feature of all the conditions and strains used in the study (Table 1). Pathways of ATP and NADH production (Citrate cycle; 2-Oxocarboxylic acid metabolism; Oxidative phosphorylation; Pentose phosphate pathway) are also found as well as those linked to translation such as amino acid metabolism (Valine, leucine and isoleucine biosynthesis; Alanine, aspartate and glutamate metabolism), and ‘ribosome’. These pathways comprise housekeeping functions necessary for cells to grow and reflect the common features of all the samples. The pathway ‘Photosynthesis-antenna proteins’ is found in the balance state although some of the samples are grown in the dark, which is not surprising as cells grown in the dark synthesize chlorophyll and assemble photosystems [28]. Some of the pathways, like ‘ribosome’ are also described in [7] in the balance state of human cells.

**Table 1:** KEGG pathways contributing most to the balance state.

KEGG pathways	Average *P*_0_	SD *P*_0_	Average *N*_*0*_	SD *N*_*0*_
Ribosome	0	0	7.21E-04	7.03E-06
Photosynthesis—antenna proteins	0	0	3.78E-04	1.02E-05
Oxidative phosphorylation	0	0	3.09E-04	2.22E-06
Phagosome	0	0	2.95E-04	2.01E-06
Glyoxylate and dicarboxylate metabolism	0	0	2.52E-04	2.59E-06
Citrate cycle (TCA cycle)	0	0	2.38E-04	2.84E-06
Valine, leucine and isoleucine biosynthesis	0	0	2.30E-04	3.08E-06
2-Oxocarboxylic acid metabolism	0	0	2.09E-04	1.88E-06
Pentose phosphate pathway	0	0	1.92E-04	1.79E-06
Alanine, aspartate and glutamate metabolism	0	0	1.89E-04	2.07E-06

*P*_0_: Positive weight of the gene set in the balance state. *N*_0_: Negative weight of the gene set in the balance state. SD: Standard deviation. See Methods for more details about the methodology.

The 10 gene pathways contributing the most to the agar versus liquid phenotype (first constraint) are listed in Tables 2 and 3 respectively. The agar-grown condition is more stressful than the liquid-grown condition since pathways such as ‘regulation of autophagy’, ‘sphingolipid metabolism’ and ‘ubiquitin mediated proteolysis’ are at the top of the list in Table 2.

**Table 2:** KEGG pathways contributing most to the agar-grown phenotype.

KEGG pathways	Average *P*_1_	SD *P*_1_	Average *N*_1_	SD *N*_1_	Average SR
Regulation of autophagy	1.03E-06	1.69E-06	6.42E-05	9.93E-06	0.02
Sphingolipid metabolism	1.81E-06	8.73E-07	5.20E-05	9.24E-06	0.03
Folate biosynthesis	1.47E-06	5.76E-07	1.80E-05	4.61E-06	0.08
Ubiquitin mediated proteolysis	2.96E-06	6.95E-07	3.11E-05	2.71E-06	0.10
Arachidonic acid metabolism	1.17E-05	3.77E-06	1.15E-04	1.75E-05	0.10
Basal transcription factors	1.74E-06	9.97E-07	1.54E-05	2.86E-06	0.11
ABC transporters	4.39E-06	1.30E-06	3.87E-05	7.76E-06	0.11
Endocytosis	4.73E-06	1.41E-06	2.49E-05	3.41E-06	0.19
SNARE interactions in vesicular transport	3.06E-06	1.86E-06	1.51E-05	2.70E-06	0.20
Sulfur relay system	3.96E-06	1.37E-06	1.95E-05	5.01E-06	0.20

*P*_1_: Positive weight of the gene set for constraint *1*, *N*_1_: Negative weight of the gene set for constraint *1*, SR: set ratios (SR = *P*_1_/*N*_1_) reflecting the contribution of the gene set to the phenotype, SD: standard deviation. See Methods more details about the methodology.

**Table 3:** KEGG pathways contributing most to the liquid-grown phenotype.

KEGG pathways	Average *P*_1_	SD *P*_1_	Average *N*_1_	SD *N*_1_	Average SR
2-Oxocarboxylic acid metabolism	9.41E-05	9.93E-06	1.99E-06	3.72E-07	47.26
Ribosome	4.22E-05	5.05E-06	1.42E-06	1.53E-06	29.61
Pentose phosphate pathway	9.72E-05	1.19E-05	3.52E-06	3.43E-06	27.59
Biosynthesis of unsaturated fatty acids	4.31E-05	5.89E-06	2.04E-06	8.55E-07	21.12
Phenylalanine, tyrosine and tryptophan biosynthesis	3.22E-05	2.93E-06	1.79E-06	2.18E-06	18.04
Proteasome	1.04E-05	4.81E-06	6.10E-07	1.01E-06	17.00
Oxidative phosphorylation	5.76E-05	5.73E-06	5.18E-06	5.17E-07	11.12
Valine, leucine and isoleucine biosynthesis	4.04E-05	4.18E-06	5.11E-06	8.26E-07	7.89
Ubiquinone and other terpenoid-quinone biosynthesis	1.41E-05	3.57E-06	1.94E-06	9.05E-07	7.31
Carbon fixation in photosynthetic organisms	7.80E-05	9.34E-06	1.09E-05	2.25E-06	7.17

*P*_1_: Positive weight of the gene set for constraint 1, *N*_1_: Negative weight of the gene set for constraint *α* = 1, SR: set ratios (SR = *P*_1_/*N*_1_) reflecting the contribution of the gene set to the phenotype, SD: standard deviation. See Methods more details about the methodology.

A few pathways of the liquid phenotype (Table 3) such as ‘2-Oxocarboxylic acid metabolism’ and ‘Oxidative phosphorylation’ are also present in the balance state, which reflects that the agar-liquid perturbation affects housekeeping genes also found in G_i0_. They reflect that the samples from the liquid medium are metabolically active and rely on ATP and NADH production linked to acetate assimilation. For the specific pathways of the liquid phenotype, one can note the presence of ‘Biosynthesis of unsaturated fatty acids’, which could indicate that the fatty acid composition of the membrane of the cells grown in liquid medium is different from that of cells grown on agar.

The second constraint allows identifying the phenotype corresponding to dark-light conditions. From the G_i2_ values computed for each transcript using surprisal analysis, the ten pathways contributing the most to the expression levels of the dark grown samples are shown in Table 4 and those contributing the most to the light-grown samples are shown in Table 5.

**Table 4:** Top 10 KEGG pathways most enriched in dark-grown samples.

KEGG pathways	Average *P*_2_	SD *P*_2_	Average *N*_2_	SD *N*_2_	Average SR
Valine, leucine and isoleucine biosynthesis	7.93E-07	6.22E-07	9.42E-05	1.34E-05	0.01
Steroid biosynthesis	5.45E-07	2.40E-06	6.40E-05	1.82E-05	0.01
Sulfur metabolism	1.40E-06	6.65E-07	7.16E-05	7.65E-06	0.02
Aminoacyl-tRNA biosynthesis	1.95E-06	1.19E-06	8.60E-05	1.10E-05	0.02
ABC transporters	1.62E-06	1.34E-06	6.31E-05	1.50E-05	0.03
Regulation of autophagy	2.06E-06	5.42E-06	6.75E-05	1.43E-05	0.03
Sphingolipid metabolism	1.91E-06	8.15E-07	3.28E-05	7.06E-06	0.06
SNARE interactions in vesicular transport	2.12E-06	1.39E-06	3.63E-05	6.83E-06	0.06
RNA transport	2.66E-06	2.57E-06	4.48E-05	5.93E-06	0.06
Arachidonic acid metabolism	8.95E-06	2.32E-06	1.41E-04	3.84E-05	0.06

*P*_2_: Positive weight of the gene set for constraint 2, *N*_2_: Negative weight of the gene set for constraint *α* = 2, SR: set ratios (SR = *P*_2_/*N*_2_) reflecting the contribution of the gene set to the phenotype, SD: standard deviation. See Methods more details about the methodology.

**Table 5:** Top 10 KEGG pathways most enriched in light grown samples.

KEGG pathways	Average *P*_*2*_	SD *P*_*2*_	Average *N*_*2*_	SD *N*_*2*_	Average SR
Photosynthesis—antenna proteins	7.93E-07	6.22E-07	9.42E-05	1.34E-05	48.36
Photosynthesis	5.45E-07	2.40E-06	6.40E-05	1.82E-05	21.47
Plant hormone signal transduction	1.40E-06	6.65E-07	7.16E-05	7.65E-06	9.43
Glycolysis / Gluconeogenesis	1.95E-06	1.19E-06	8.60E-05	1.10E-05	7.04
Amino sugar and nucleotide sugar metabolism	1.62E-06	1.34E-06	6.31E-05	1.50E-05	5.57
Citrate cycle (TCA cycle)	2.06E-06	5.42E-06	6.75E-05	1.43E-05	5.26
Fructose and mannose metabolism	1.91E-06	8.15E-07	3.28E-05	7.06E-06	4.03
Pentose phosphate pathway	2.12E-06	1.39E-06	3.63E-05	6.83E-06	3.84
Nitrogen metabolism	2.66E-06	2.57E-06	4.48E-05	5.93E-06	3.51
Carbon fixation in photosynthetic organisms	8.95E-06	2.32E-06	1.41E-04	3.84E-05	3.15

*P*_*2*_: Positive weight of the gene set for constraint *2*, *N*_*2*_: Negative weight of the gene set for constraint *2*, SR: set ratios (SR = P_2_/N_2_) reflecting the contribution of the gene set to the phenotype, SD: standard deviation. See Methods more details about the methodology.

Some of the gene sets characterizing the dark phenotype (Table 4) are common with the agar phenotype (Table 2) (ABC transporters, Regulation of autophagy, Sphingolipid metabolism, SNARE interactions in vesicular transport, Arachidonic acid metabolism) although their order of importance is not the same as for the agar-grown samples. The second gene set prevailing most in the dark phenotype (Table 4) is ‘Steroid biosynthesis’. Interestingly it has been reported that the lack of ergosterol in yeasts, a sterol found in membranes of *Chlamydomonas* [29], impairs growth on respiratory substrates [30]. Thus the presence of this pathway could suggest membrane adaptation in dark-grown samples. In addition, the third pathway prevailing most in the dark, ‘sulfur metabolism’, could indicate that dark-grown samples may suffer from sulfur limitation.

Logically, pathways of light utilization (Photosynthesis—antenna proteins; Photosynthesis) in light-grown phenotype (Table 5) are in the first top two pathways in addition to the pathways of ATP and NADH production that could be linked to acetate utilization such as ‘Glycolysis / Gluconeogenesis’; ‘Citrate cycle’, ‘Pentose phosphate pathway’. Some pathways of Table 5 are also found in the balance state, which reflects that the dark-light perturbation affects housekeeping genes also found in G_i0_.

In summary, our comparative analysis of the pathways of G_i2_ suggests that the dark phenotype is more stressful than the light growth mode in the tested conditions. The same conclusion was also found below when comparing the agar versus liquid growth mode above. Therefore, even though the samples were analyzed when the number of cells per unit (ml or colony) was roughly the same (between 5x10^5^ and 5x10^6^ cells), it is clear that the agar and the dark growth modes were not optimized in terms of cultivation.

### Analysis of the 100 genes contributing the most to agar/liquid and dark/light phenotype

#### First constraint

**Fig 3:**
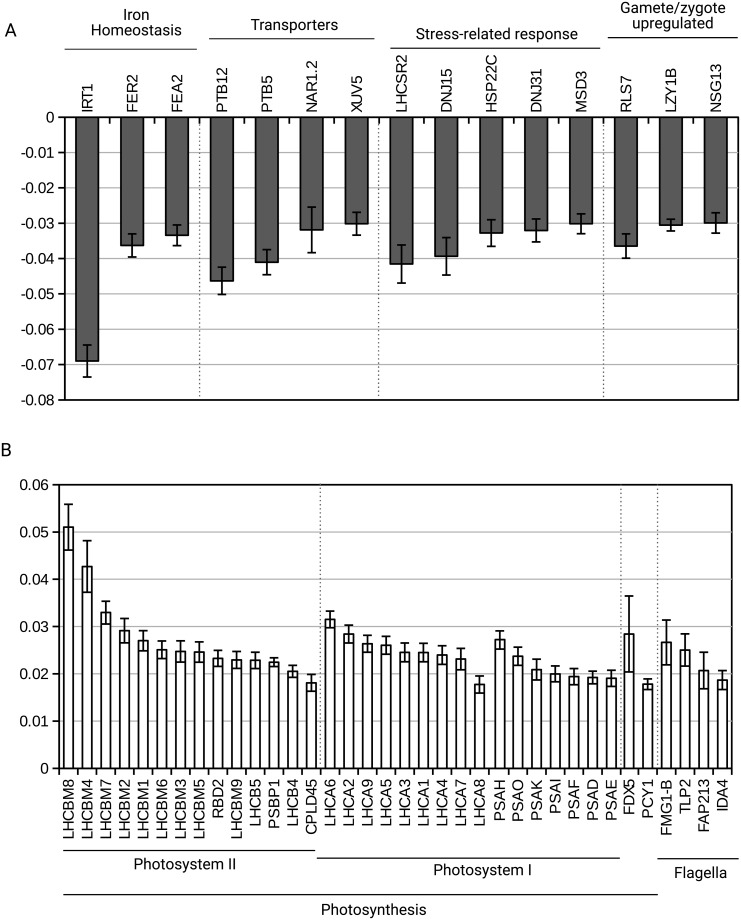
Genes with identified function among the first 100 genes in agar- and liquid- grown samples. (A) agar-grown samples. (B) liquid-grown samples.

In addition to ranking KEGG pathways according to their G_i1_ and G_i2_ values to define the biological pathways most important for a specific phenotype, it is also possible to quantify which individual genes contribute most to the phenotypes. Most of the 100 genes that significantly contribute to the phenotype of the agar-grown samples have unknown function (S3 Fig). Some of those with identified functions could be grouped into categories (Fig 3A). Transcripts related to Fe (IRT1, FER2, FEA2) limitation are found with IRT1 (iron-nutrition responsive ZIP transporter family) at the very top of the list of the first 100 most contributing genes (S3 Fig) for the agar-grown phenotype. We can also notice transcripts encoding various transporters: members of the PTB family (PTB12, 5), for PO_4_^3-^ (P_i_) uptake coupled with Na^+^ transport, and others transporters (NAR1.2 for nitrate, XUV5 for xanthine, uracil, vitamin C). The increased weight of these transcripts could indicate limitations in P_i_ and Fe in the agar-grown samples. As a matter of fact, the *LHCSR2* transcript encoding an antenna protein activated upon excess of light, iron, copper, and phosphate deficiencies [31–34], is found as well as *MSD3*. This gene encodes Mn superoxide dismutase whose transcription is increased upon iron deficiency [35]. At last, transcripts specific of gamete/zygote are found [36], which could also indicate that the agar-type of growth represents a stressful condition, where the process of sexual differentiation starts. In conclusion, this analysis suggests that samples grown on agar suffer from nutrient deficiency which in turns provokes the activation of stress-related genes in the conditions tested. These results suggest that the colonies already suffered from nutrient deficiency when they were picked up (10 days growth in the light or 3 weeks growth in the dark). This implies that an optimization of the cultivation medium in terms of iron and phosphate concentrations could be useful to improve growth on agar.

For the liquid-grown samples (Fig 3B and S4 Fig), many transcripts of light-harvesting complex I (LHCA1, 2, 3, 4, 5, 6, 7 and 8) and complex II (LHCBM1, 2, 3, 4, 5, 6, 7, 8 and 9, LHCB4, 5) are present.

#### Second constraint

**Fig 4:**
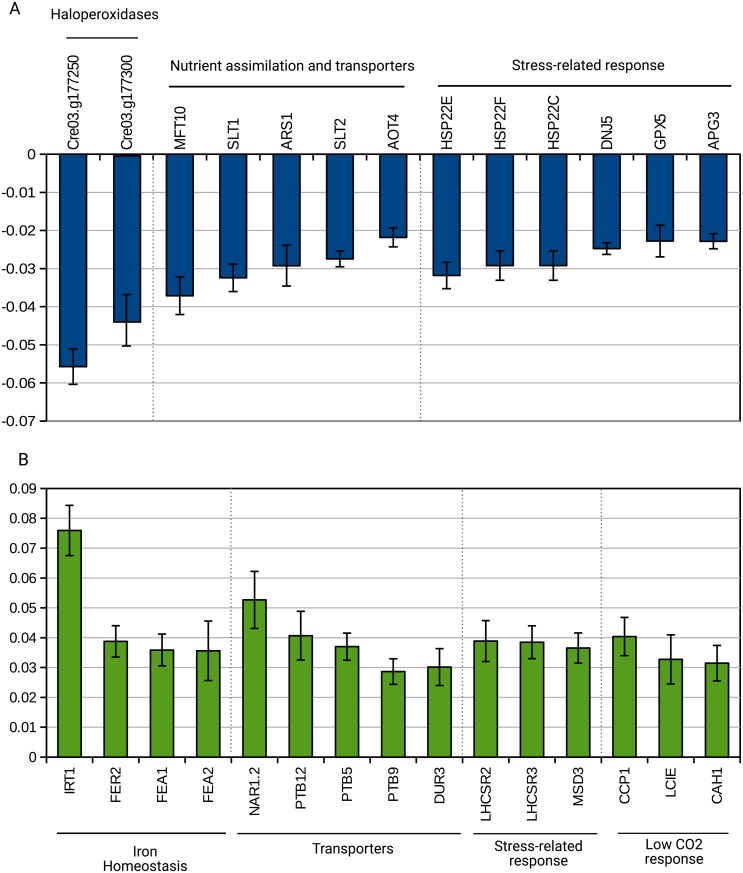
Genes with identified function among the first 100 genes in dark- and light- grown samples. (A) dark-grown samples. (B) light-grown samples.

Fig 4 describes transcripts with assigned function amongst the first 100 genes contributing the most to the dark-grown phenotype (Fig 4A) and to the light-phenotype (Fig 4B) (the complete list of the first 100 genes is found in S5 and S6 Figs). Two genes encoding proteins with putative haloperoxidase activity (Cre03.g177250 and Cre03.g177300) are found at the very top of the list of the 100 genes for the dark-grown phenotype. In addition, stress related genes are found: the transcripts of three isoforms of the heat shock protein HSP22 contribute to the dark-grown phenotype (Fig 4A). The HSP22C isoform is targeted to mitochondria while HSP22E and F are targeted to chloroplast when using Predalgo for prediction [37], suggesting proteins modifications inside the chloroplast and mitochondria. Transcripts encoding transporters are also found important to describe the dark-grown samples. Indeed, transcripts encoding transporters for SO_4_^2-^ are found (SLT1 and SLT2) as well as extracellular arylsulfatase (ARS1), which is typical of cells experiencing low SO_4_^2-^ availability [38], as also pointed out by the pathway analysis where sulfur metabolism is found important for the dark-grown samples.

The light-grown phenotype (Fig 4B) is characterized by transcripts encoding proteins involved in iron homeostasis, transporters, stress-related response and low CO_2_ availability, which is typical of air-grown cultures where CO_2_ is limiting [39]. In conclusion, the analysis of the individual genes of the second constraint suggests that the cultivation medium could be improved in terms of SO_4_ or CO_2_ availability for the dark-grown samples and the light-grown samples respectively.

Photosynthesis genes are thus predominant in the liquid phenotype (Fig 3B and S4 Fig), much more than in the light phenotype (Fig 4B and S5 Fig). We looked back at the raw data of the genes involved in photosynthesis in the top 100 genes most important for the liquid phenotype (S4 Fig) and report in S7 Fig the log_2_ FPKM values of these genes in the different types of samples (AL, AD, LL, LD). All of the photosynthetic transcripts are in higher amounts in the conditions liquid@light (LL) and liquid@dark (LD) compared to agar@light (AL) and agar@dark (AD). These results are thus in agreement with the surprisal analysis results, which highlights these genes as important to explain the liquid phenotype, irrespective of the presence or absence of light. This suggests that the photosynthetic genes are not upregulated in AL samples as much as in the LL samples because the cells forming the colonies are not all photosynthesizing: cells at the surface are exposed to the light but cells inside the colonies do not receive or receive less light. A part of the cells of the colonies probably turn to a heterotrophic growth mode and become stressed because of depletion of acetate and other nutrients. Concerning the presence of the photosynthetic transcripts in the LD condition, it is established that chlorophylls are synthesized in the dark [23,28], which goes hand in hand with the presence of the transcripts encoding proteins associated with them (LHCA and LHCB) and transcripts encoding structural proteins of PSI and PSII.

### Comparison of the surprisal and statistical analysis phenotype characterization

**Fig 5:**
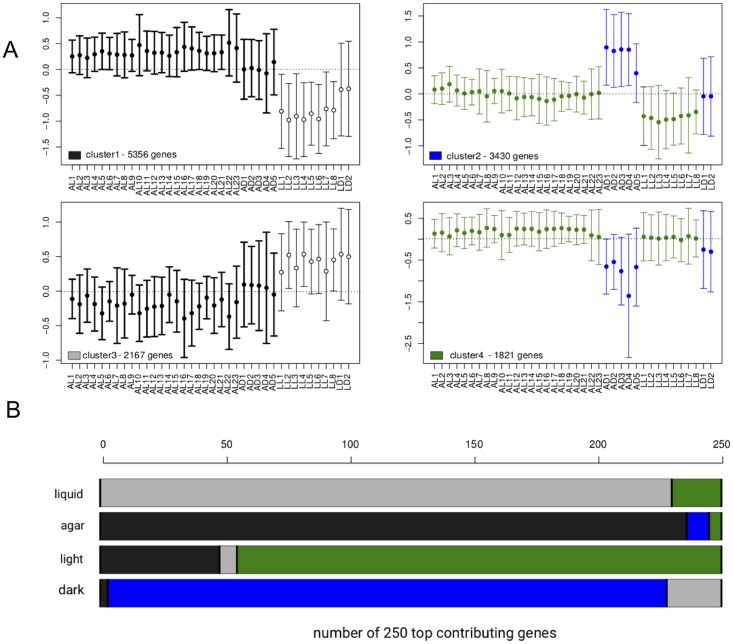
Comparison of top-contributing genes according to surprisal analysis and K-means clustering of transcripts. (A) Centroid plots with mean and standard deviation of the expression values [Ln(meancenteredFPKM)] of the different genes belonging to the different clusters for each sample. Agar-upregulated (cluster 1, black), dark-upregulated (cluster 2, blue), light-upregulated (cluster 3, grey) and liquid-upregulated (cluster 4, green). (B) Cumulative barplot describing how many of the 250 most contributing genes to the different phenotypes described according to the first 2 constraints (see Fig 1) belong to the 4 different clusters according to K-means clustering. The same color code as in (A) is used for representing the samples corresponding to the different phenotypes.

We also tested whether K-means clustering of expression values as implemented in the MeV software package [25] could obtain a similar separation of phenotypes (Fig 5). Cluster analysis on mean centered ln(FPKM) values of the 38 samples is statistically meaningful for four groups. The four groups (Fig 5A) correspond to agar upregulated (cluster 1, black), dark-upregulated (cluster 2, blue), light-upregulated (cluster 3, grey) and liquid-upregulated (cluster 4, green). The top 250 genes contributing most to a specific phenotype determined by the surprisal analysis correspond for more than 80% to clusters corresponding to the same phenotype (Fig 5B) determined by K-means clustering. K-means clustering and surprisal analysis therefore leads to similar phenotypes. The difference is that surprisal analysis provides a thermodynamical analysis of the phenotypes, that are characterized by the changes that they induce on the free energy compared to the balance state. K-means clustering results in mutually exclusive lists, while for surprisal analysis the constraints describe the contribution of all genes to that constraint, but with different weights (the values of *G*_*iα*_, see Material and methods).

We also compared the top 250 contributing genes to the first two constraints with the significantly differentially expressed genes obtained by Differential Gene Expression (DGE) using an EdgeR test [24] as implemented by CLC Genomics Workbench (S8 Fig). Overall, we get a large overlap between the top 250 genes that characterized phenotypes identified in surprisal analysis and the differentially expressed genes of the corresponding growth conditions.

## Conclusions

Surprisal analysis of transcriptomics data obtained from *Chlamydomonas* samples cultivated in four different conditions (agar@dark, agar@light, liquid@dark, liquid@light) and in two different laboratories identifies two specific constraints that disentangle the effects on the gene expression levels of the agar/liquid and dark/light conditions. The meta-analysis of the phenotypes identified by the constraints resolve the biological processes specifically activated under these two different sets of conditions. Biological processes are resolved by pathway analysis which is efficient, but has some limitations as only 3145 genes on the 15,143 protein-coding gene predictions [2] are categorized into KEGG pathways. Thus, the individual list of the 100 genes contributing most to a phenotype is also a valuable tool to understand the constraints. As a matter of fact, the two genes Cre03.g177250 and Cre03.g177300 encoding putative haloperoxidase do not fall into any KEGG pathway. Comparison between results obtained by surprisal analysis with those obtained by purely classical approaches (K-means clustering and DGE analysis) concur with the results of the surprisal analysis.

In conclusion, our results open the way to a more detailed characterization of the less-studied modes of growth, dark and agar, which are emerging as promising for biotechnological purposes in the field of solid-state photobioreactors and growth in fermenters. We show that these two conditions are more stressful than light or liquid cultivation modes in the tested experimental setups. One would now aim to find experimental setups where parameters like medium composition could be modified in such a way that these two conditions could not be differentiated from the light and liquid modes on the level of nutrient related pathways and genes. In addition, as we have demonstrated that the pipeline developed for the analysis of *Chlamydomonas* gene expression by surprisal analysis can be used on data sets coming from different laboratories and reference strains, we are confident that our method could be a method of choice in future investigations aiming at disentangling specific constraints and phenotypes from large data sets of different origins.

## Supporting information

S1 Fig Agar-grown samples in the light. The microalgal cells form colonies. RNA seq analysis was performed on the colonies that were numbered.
(DOCX)

S2 Fig Lagrange multipliers values *λ*_3_(*s*) of the third constraint. (DOCX)

S3 Fig Distribution of G_1_ values of the first hundred genes contributing most to the agar-grown samples. Data are computed from 1000 random combinations of 14 samples.
(DOCX)

S4 Fig Distribution of G_1_ values of the first hundred genes contributing most to the liquid-grown samples. Data are computed from 1000 random combinations of 14 samples.
(DOCX)

S5 Fig Distribution of G_2_ values of the first hundred genes contributing most to the dark-grown samples. Data are computed from 1000 random combinations of 14 samples.
(DOCX)

S6 Fig Distribution of G_2_ values of the first hundred genes contributing most to the light-grown samples. Data are computed from 1000 random combinations of 14 samples.
(DOCX)

S7 Fig Log_2_(FPKM) values calculated on the FPKM values for the photosynthetic genes identified in S4 Fig in the AL, AD, LL and LD samples. LHCA genes: light-harvesting complex I; LHCB: light-harvesting complex II, PS: photosynthetic genes (PSAH-O-K-F-D-5: PSI; PSBP1: PSII).
(DOCX)

S8 Fig Comparison of 250 top-contributing genes according to surprisal analysis and differential gene expression analysis (DGE). The columns denote the number of top-contributing genes according to surprisal analysis which are also present among the significantly differentially expressed genes upregulated in respectively dark, light, liquid and agar as obtained by the two available pairwise DGE comparisons.
(DOCX)

S1 Table Total sequenced reads and reads left after trimming and filtering for samples grown on agar in the light (AL1-AL23). (DOCX)

S2 Table Total sequenced reads and reads left after trimming and filtering for samples grown on agar in the dark (AD1-AD5). (DOCX)

S3 Table Total sequenced reads and reads left after trimming and filtering for samples grown in liquid and in the light (LL1-LL8). Sequencing yield for 3 biological replicates per time is reported. Replicates were mapped to the *Chlamydomonas* genome and average expression levels were calculated.
(DOCX)

S4 Table Total sequenced reads [23] and reads left after trimming and filtering for samples grown in liquid medium and in the dark (LD1-LD2). (DOCX)

S5 Table FPKM values for the 12774 transcripts identified in the 38 samples. Values lower than 0.01 FPKM were substituted with 0.01 FPKM to allow the computation of logarithms and expression ratios.
(CSV)

S6 Table Wilcoxon t-test on λα values on surprisal analysis on all samples, testing for significant differences between dark and light- grown samples. Note that only the first and second constraint have P-values < 0.001 when comparing the phenotypes for respectively the medium and light regime (indicated with an asterisk).
(DOCX)

S7 Table KEGG pathways in *Chlamydomonas*. The number of genes in the pathways and in our analysis is indicated.
(DOCX)
