# Supplementary material for: Surprisal analysis of genome-wide transcript profiling identifies differentially expressed genes and pathways associated with four growth conditions in the microalga Chlamydomonas
Source: PLoS One. 2018 Apr 17;13(4):e0195142. doi: 10.1371/journal.pone.0195142 (PMC5903653; doi:10.1371/journal.pone.0195142)
Supplement: S1 Fig — The microalgal cells form colonies. RNA seq analysis was performed on the colonies that were numbered. (DOCX) [file pone.0195142.s001.docx]

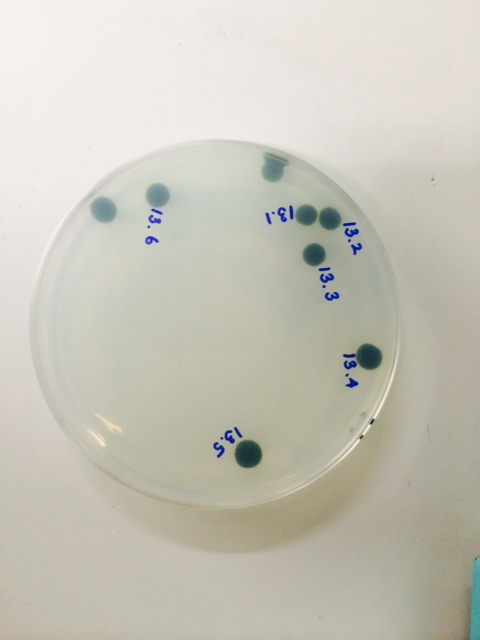


**S1 Fig. Agar-grown samples in the light.** The microalgal cells form colonies. RNA seq analysis was performed on the colonies that were numbered.
