# Supplementary figures and images for: Surprisal analysis of genome-wide transcript profiling identifies differentially expressed genes and pathways associated with four growth conditions in the microalga Chlamydomonas
Source: PLoS One. 2018 Apr 17;13(4):e0195142. doi: 10.1371/journal.pone.0195142 (PMC5903653; doi:10.1371/journal.pone.0195142)

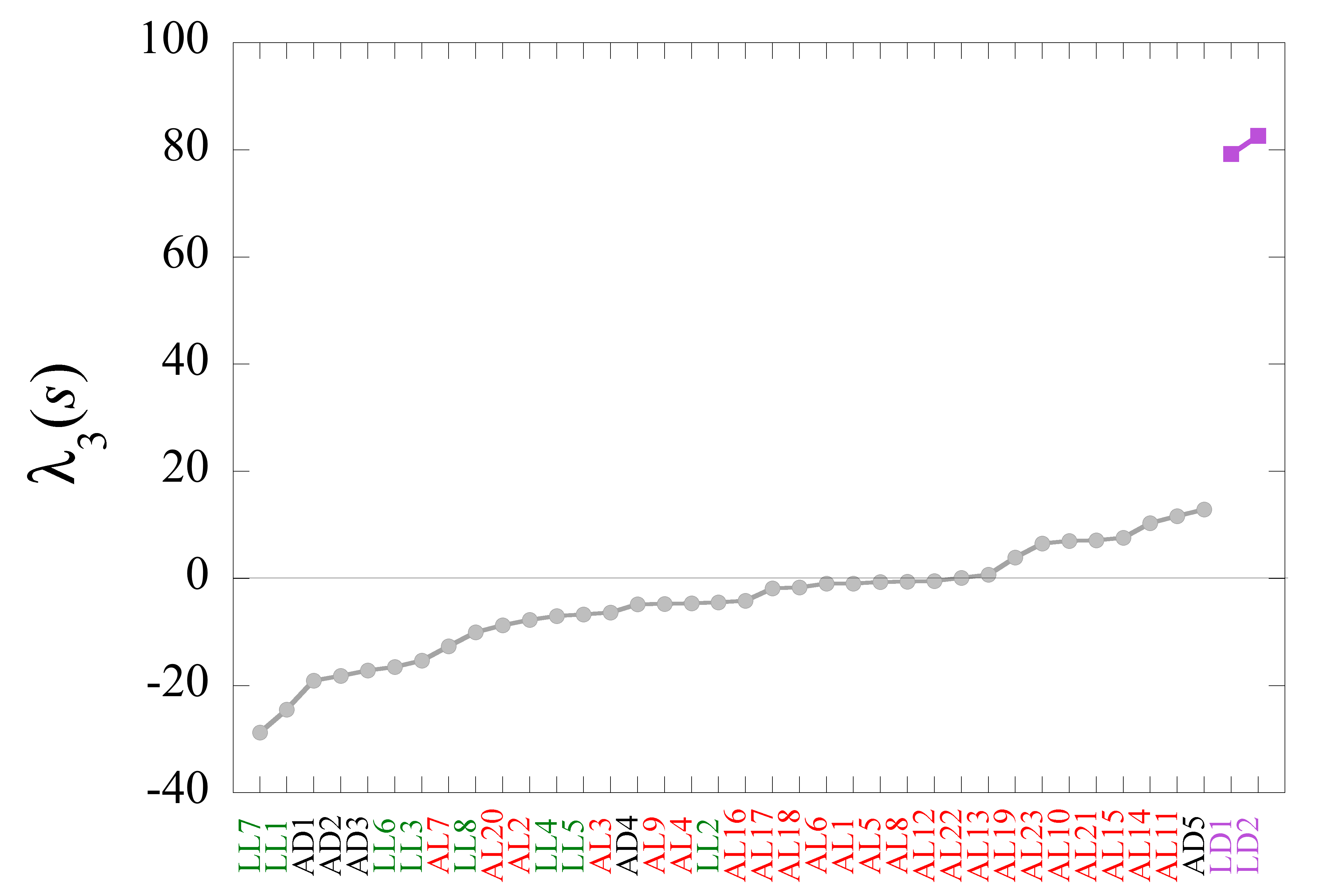


**S2 Fig**. **Values of the Lagrange multipliersof the third constraint.**

Supplement: S2 Fig — (DOCX) [file pone.0195142.s002.docx]
