# Supplementary material for: Surprisal analysis of genome-wide transcript profiling identifies differentially expressed genes and pathways associated with four growth conditions in the microalga Chlamydomonas
Source: PLoS One. 2018 Apr 17;13(4):e0195142. doi: 10.1371/journal.pone.0195142 (PMC5903653; doi:10.1371/journal.pone.0195142)
Supplement: S4 Fig — Data are computed from 1000 random combinations of 14 samples. (DOCX) [file pone.0195142.s004.docx]

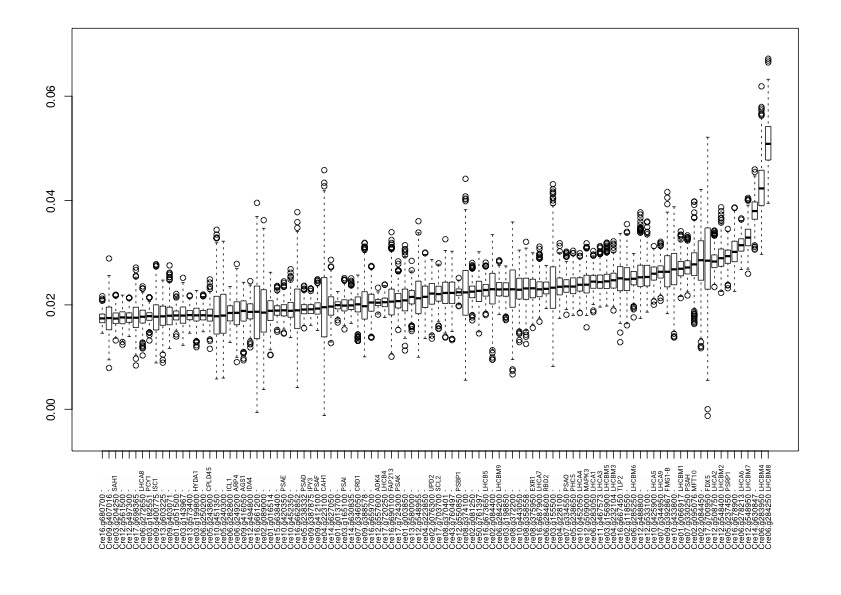


**S4 Fig. Distribution of G_1_ values of the first hundred genes contributing most to the liquid-grown samples.** Data are computed from 1000 random combinations of 14 samples.
