# Supplementary material for: Surprisal analysis of genome-wide transcript profiling identifies differentially expressed genes and pathways associated with four growth conditions in the microalga Chlamydomonas
Source: PLoS One. 2018 Apr 17;13(4):e0195142. doi: 10.1371/journal.pone.0195142 (PMC5903653; doi:10.1371/journal.pone.0195142)
Supplement: S7 Fig — LHCA genes: light-harvesting complex I; LHCB: light-harvesting complex II, PS: photosynthetic genes (PSAH-O-K-F-D-5: PSI; PSBP1: PSII). (DOCX) [file pone.0195142.s007.docx]

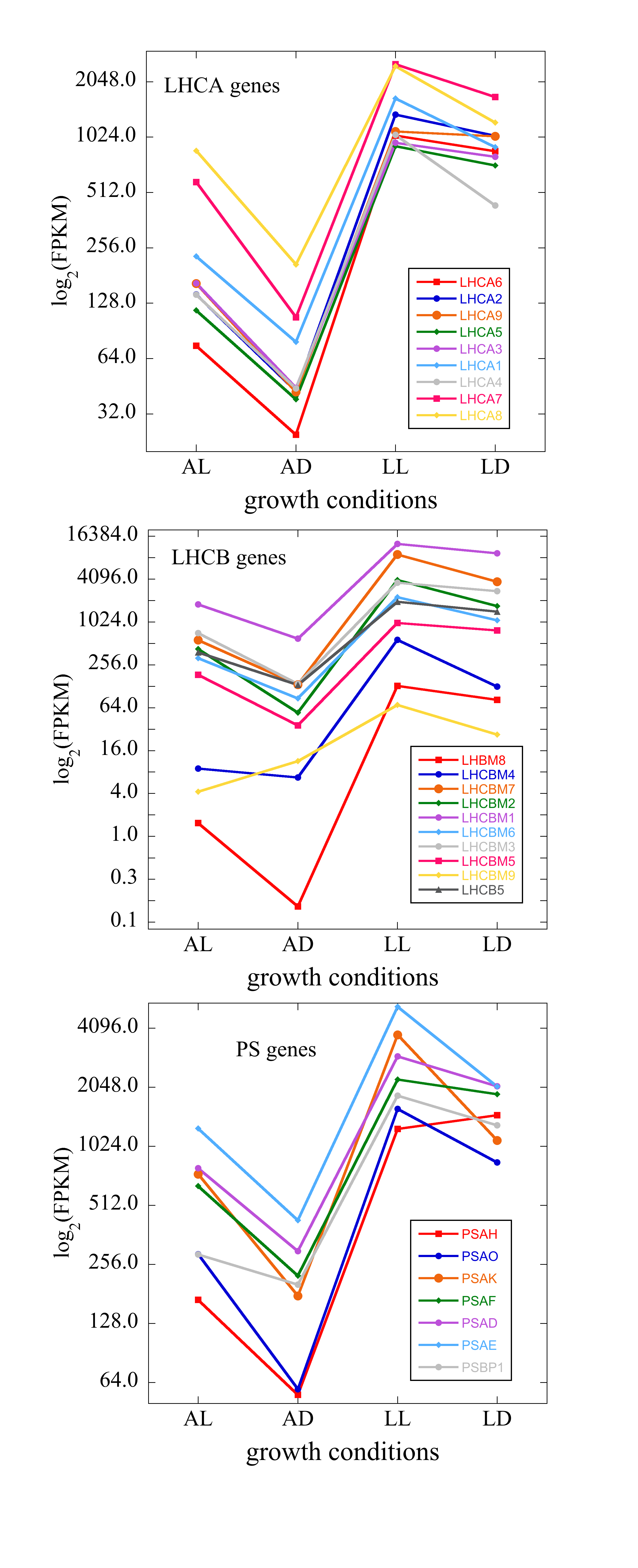


**S7 Fig.** **Log_2_(FPKM) values calculated on the FPKM values for the photosynthetic genes identified in S4 Fig in the AL, AD, LL and LD samples**. LHCA genes: light-harvesting complex I; LHCB: light-harvesting complex II, PS: photosynthetic genes (PSAH-O-K-F-D-5: PSI; PSBP1: PSII)
