# Supplementary material for: Surprisal analysis of genome-wide transcript profiling identifies differentially expressed genes and pathways associated with four growth conditions in the microalga Chlamydomonas
Source: PLoS One. 2018 Apr 17;13(4):e0195142. doi: 10.1371/journal.pone.0195142 (PMC5903653; doi:10.1371/journal.pone.0195142)
Supplement: S8 Fig — The columns denote the number of top-contributing genes according to surprisal analysis which are also present among the significantly differentially expressed genes upregulated in respectively dark, light, liquid and agar as obtained by the two available pairwise DGE comparisons. (DOCX) [file pone.0195142.s008.docx]

**
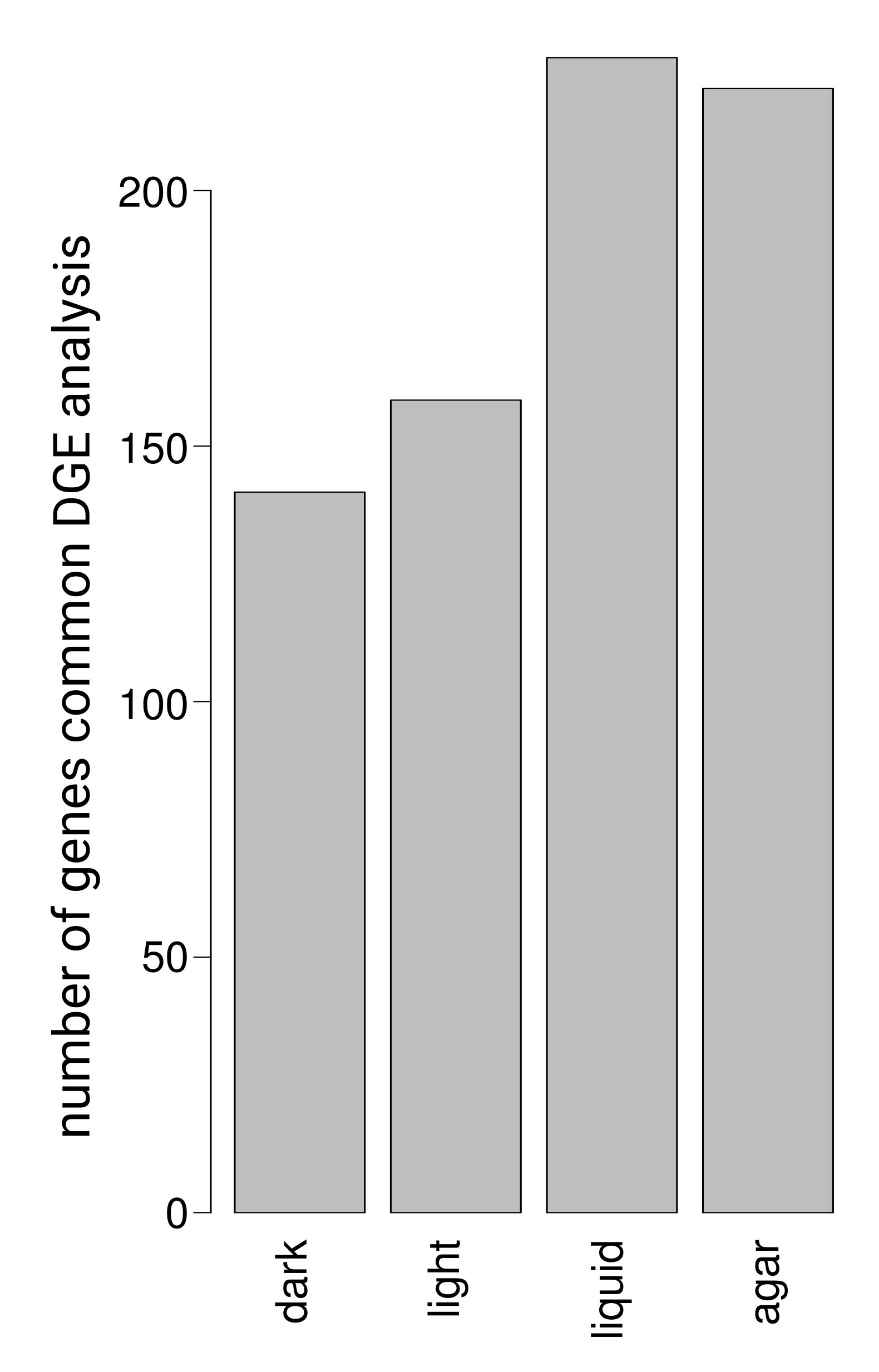
**

**S8 Fig. Comparison of 250 top-contributing genes according to surprisal analysis and differential gene expression analysis (DGE).** The columns denote the number of top-contributing genes according to surprisal analysis which are also present among the significantly differentially expressed genes upregulated in respectively dark, light, liquid and agar as obtained by the two available pairwise DGE comparisons.
