# Supplementary material for: Surprisal analysis of genome-wide transcript profiling identifies differentially expressed genes and pathways associated with four growth conditions in the microalga Chlamydomonas
Source: PLoS One. 2018 Apr 17;13(4):e0195142. doi: 10.1371/journal.pone.0195142 (PMC5903653; doi:10.1371/journal.pone.0195142)
Supplement: S1 Table — (DOCX) [file pone.0195142.s009.docx]

**S1 Table**. **Total sequenced reads and reads left after trimming and filtering for samples grown on agar in the light (AL1-AL23).**

| **Plate** | **Colony** | **Sample** | **Sequenced reads** | **Trimmed and Filtered** |
| --- | --- | --- | --- | --- |
| 13 | 1 | AL1 | 28,118,861 | 21,191,420 |
| 13 | 4 | AL2 | 54,018,347 | 39,273,282 |
| 13 | 5 | AL3 | 28,462,165 | 21,939,202 |
| 14 | 2 | AL4 | 38,411,025 | 28,519,128 |
| 14 | 3 | AL5 | 26,920,899 | 20,544,469 |
| 14 | 5 | AL6 | 53,059,482 | 38,508,905 |
| 15 | 2 | AL7 | 54,917,685 | 39,651,983 |
| 15 | 3 | AL8 | 27,266,476 | 20,425,021 |
| 15 | 4 | AL9 | 58,192,765 | 41,876,150 |
| 16 | 1 | AL10 | 30,370,200 | 22,761,206 |
| 16 | 2 | AL11 | 28,151,015 | 21,410,571 |
| 17 | 1 | AL12 | 58,461,396 | 41,715,326 |
| 17 | 2 | AL13 | 70,264,928 | 50,545,301 |
| 17 | 4 | AL14 | 30,712,346 | 23,525,592 |
| 18 | 1 | AL15 | 58,616,241 | 42,321,516 |
| 18 | 2 | 18-2* | 12,989,723 | 9,882,036 |
| 18 | 3 | AL16 | 27,186,167 | 20,340,510 |
| 18 | 4 | AL17 | 29,511,161 | 21,935,756 |
| 19 | 2 | AL18 | 29,328,182 | 22,536,454 |
| 19 | 3 | AL19 | 25,936,016 | 19,397,612 |
| 19 | 4 | AL20 | 56,482,051 | 39,469,659 |
| 19 | 5 | AL21 | 23,932,218 | 18,029,826 |
| 24 | 6 | AL22 | 34,113,152 | 26,032,413 |
| 24 | 8 | AL23 | 35,681,909 | 28,103,594 |
