# Supplementary material for: Surprisal analysis of genome-wide transcript profiling identifies differentially expressed genes and pathways associated with four growth conditions in the microalga Chlamydomonas
Source: PLoS One. 2018 Apr 17;13(4):e0195142. doi: 10.1371/journal.pone.0195142 (PMC5903653; doi:10.1371/journal.pone.0195142)
Supplement: S2 Table — (DOCX) [file pone.0195142.s010.docx]

**S2 Table**. **Total sequenced reads and reads left after trimming and filtering for samples grown on agar in the dark (AD1-AD5).**

| **Plate** | **Colony** | **Sample** | **Sequenced** | **Trimmed and Filtered** |
| --- | --- | --- | --- | --- |
| DP3 | 1 | AD1 | 44,118,285 | 38,866,029 |
| DP4 | 2 | AD2 | 48,673,347 | 42,807,961 |
| DP4 | 3 | AD3 | 34,181,742 | 29,780,235 |
| DT1 | 1 | AD4 | 39,009,729 | 34,659,333 |
| DT2 | 1 | AD5 | 35,570,728 | 31,042,932 |
