# Supplementary material for: Surprisal analysis of genome-wide transcript profiling identifies differentially expressed genes and pathways associated with four growth conditions in the microalga Chlamydomonas
Source: PLoS One. 2018 Apr 17;13(4):e0195142. doi: 10.1371/journal.pone.0195142 (PMC5903653; doi:10.1371/journal.pone.0195142)
Supplement: S3 Table — Sequencing yield for 3 biological replicates per time is reported. Replicates were mapped to the Chlamydomonas genome and average expression levels were calculated. (DOCX) [file pone.0195142.s011.docx]

**S3 Table. Total sequenced reads and reads left after trimming and filtering for samples grown in liquid and in the light (LL1-LL8).** Sequencing yield for 3 biological replicates per time is reported. Replicates were mapped to the *Chlamydomonas* genome and average expression levels were calculated**.**

| **Acetate concentration (mM)** | **Time (h)** | **Replicate** | **Sample** | **Sequenced** | **Trimmed and Filtered** |
| --- | --- | --- | --- | --- | --- |
| 17 | 12 | 1 | LL1 | 27,596,703 | 25,600,013 |
| 17 | 12 | 2 | LL1 | 30,114,986 | 28,063,350 |
| 17 | 12 | 3 | LL1 | 28,741,589 | 26,807,561 |
| 17 | 28 | 1 | LL2 | 24,652,570 | 24,652,570 |
| 17 | 28 | 2 | LL2 | 26,249,741 | 26,249,741 |
| 17 | 28 | 3 | LL2 | 24,994,681 | 24,994,681 |
| 31 | 12 | 1 | LL3 | 19,457,776 | 19,695,743 |
| 31 | 12 | 2 | LL3 | 19,968,434 | 18,453,200 |
| 31 | 12 | 3 | LL3 | 19,268,571 | 19,452,219 |
| 31 | 28 | 1 | LL4 | 20,819,017 | 18,047,009 |
| 31 | 28 | 2 | LL4 | 19,597,169 | 18,541,383 |
| 31 | 28 | 3 | LL4 | 20,640,312 | 17,952,421 |
| 44 | 12 | 1 | LL5 | 21,981,546 | 20,759,397 |
| 44 | 12 | 2 | LL5 | 19,914,125 | 18,779,332 |
| 44 | 12 | 3 | LL5 | 19,618,977 | 18,322,514 |
| 44 | 28 | 1 | LL6 | 20,092,783 | 18,797,355 |
| 44 | 28 | 2 | LL6 | 20,687,575 | 19,400,482 |
| 44 | 28 | 3 | LL6 | 20,140,363 | 18,784,045 |
| 57.5 | 12 | 1 | LL7 | 18,852,415 | 17,636,204 |
| 57.5 | 12 | 2 | LL7 | 19,347,156 | 18,105,831 |
| 57.5 | 12 | 3 | LL7 | 20,673,419 | 19,490,295 |
| 57.5 | 28 | 1 | LL8 | 18,525,469 | 17,490,336 |
| 57.5 | 28 | 2 | LL8 | 19,408,717 | 18,295,960 |
| 57.5 | 28 | 3 | LL8 | 18,761,420 | 17,594,155 |
