# Supplementary material for: Surprisal analysis of genome-wide transcript profiling identifies differentially expressed genes and pathways associated with four growth conditions in the microalga Chlamydomonas
Source: PLoS One. 2018 Apr 17;13(4):e0195142. doi: 10.1371/journal.pone.0195142 (PMC5903653; doi:10.1371/journal.pone.0195142)
Supplement: S4 Table — (DOCX) [file pone.0195142.s012.docx]

**S4 Table. Total sequenced reads [23] and reads left after trimming and filtering for samples grown in liquid medium and in the dark (LD1-LD2).**

| **Replicate** | **Sample** | **Sequenced** | **Trimmed and Filtered** |
| --- | --- | --- | --- |
| 1 | LD1 | 26,529,796 | 24,149,153 |
| 2 | LD2 | 24,448,095 | 20,745,108 |
