# Supplementary material for: Surprisal analysis of genome-wide transcript profiling identifies differentially expressed genes and pathways associated with four growth conditions in the microalga Chlamydomonas
Source: PLoS One. 2018 Apr 17;13(4):e0195142. doi: 10.1371/journal.pone.0195142 (PMC5903653; doi:10.1371/journal.pone.0195142)
Supplement: S6 Table — Note that only the first and second constraint have P-values < 0.001 when comparing the phenotypes for respectively the medium and light regime (indicated with an asterisk). (DOCX) [file pone.0195142.s014.docx]

**S6 Table. Wilcoxon t-test on λα values on surprisal analysis on all samples, testing for significant differences between dark vs light and agar vs liquid grown samples.** Note that only the first and second constraint have P-values < 0.001 when comparing the phenotypes for respectively the medium and light regime (indicated with an asterisk)**.**

|  | **Phenotype: dark vs light** | | **Phenotype: agar vs liquid** | |
| --- | --- | --- | --- | --- |
| **constraint** | **W** | **P-value (two-sided)** | **W** | **P-value (two-sided)** |
| 1 | 56 | 0.05 | 0 | **4.00E-09*** |
| 2 | 217 | **1.58E-07*** | 150 | 0.76 |
| 3 | 107 | 0.97 | 192 | 0.09 |
| 4 | 73 | 0.19 | 98 | 0.17 |
| 5 | 145 | 0.18 | 120 | 0.52 |
| 6 | 133 | 0.37 | 150 | 0.76 |
| 7 | 118 | 0.74 | 125 | 0.64 |
| 8 | 80 | 0.30 | 156 | 0.61 |
| 9 | 119 | 0.71 | 145 | 0.88 |
| 10 | 103 | 0.85 | 104 | 0.24 |
| 11 | 77 | 0.25 | 108 | 0.30 |
| 12 | 110 | 0.97 | 153 | 0.68 |
| 13 | 123 | 0.61 | 141 | 0.99 |
| 14 | 88 | 0.46 | 141 | 0.99 |
| 15 | 111 | 0.94 | 137 | 0.94 |
| 16 | 111 | 0.94 | 134 | 0.86 |
| 17 | 103 | 0.85 | 170 | 0.33 |
| 18 | 108 | 1.00 | 136 | 0.91 |
| 19 | 102 | 0.83 | 125 | 0.64 |
| 20 | 94 | 0.61 | 156 | 0.61 |
| 21 | 109 | 1.00 | 124 | 0.61 |
| 22 | 97 | 0.69 | 139 | 0.99 |
| 23 | 109 | 1.00 | 140 | 1.00 |
| 24 | 109 | 1.00 | 131 | 0.78 |
| 25 | 118 | 0.74 | 139 | 0.99 |
| 26 | 114 | 0.85 | 138 | 0.96 |
| 27 | 124 | 0.58 | 139 | 0.99 |
| 28 | 94 | 0.61 | 109 | 0.32 |
| 29 | 121 | 0.66 | 165 | 0.42 |
| 30 | 106 | 0.94 | 152 | 0.71 |
| 31 | 104 | 0.88 | 141 | 0.99 |
| 32 | 96 | 0.66 | 146 | 0.86 |
| 33 | 116 | 0.80 | 136 | 0.91 |
| 34 | 108 | 1.00 | 164 | 0.44 |
| 35 | 112 | 0.91 | 149 | 0.78 |
| 36 | 101 | 0.80 | 137 | 0.94 |
| 37 | 111 | 0.94 | 137 | 0.94 |
