# Supplementary material for: Surprisal analysis of genome-wide transcript profiling identifies differentially expressed genes and pathways associated with four growth conditions in the microalga Chlamydomonas
Source: PLoS One. 2018 Apr 17;13(4):e0195142. doi: 10.1371/journal.pone.0195142 (PMC5903653; doi:10.1371/journal.pone.0195142)
Supplement: S7 Table — The number of genes in the pathways and in our analysis is indicated. (DOCX) [file pone.0195142.s015.docx]

**S7 Table. KEGG pathways in *Chlamydomonas***. The number of genes in the pathways and in our analysis is indicated.

| KEGG Pathway | N° genes in our analysis | Total n° genes |
| --- | --- | --- |
| Carbon_metabolism | 131 | 140 |
| Biosynthesis_of_amino_acids | 120 | 126 |
| Ribosome | 124 | 125 |
| Purine_metabolism | 108 | 115 |
| Spliceosome | 108 | 110 |
| RNA_transport | 89 | 95 |
| Protein_processing_in_endoplasmic_reticulum | 81 | 82 |
| Pyrimidine_metabolism | 79 | 82 |
| Oxidative_phosphorylation | 67 | 69 |
| Ubiquitin_mediated_proteolysis | 60 | 64 |
| RNA_degradation | 57 | 61 |
| Ribosome_biogenesis_in_eukaryotes | 58 | 61 |
| Endocytosis | 53 | 54 |
| Peroxisome | 47 | 50 |
| Pyruvate_metabolism | 46 | 49 |
| Glycolysis-Gluconeogenesis | 45 | 49 |
| mRNA_surveillance_pathway | 44 | 47 |
| Aminoacyl-tRNA_biosynthesis | 42 | 45 |
| Starch_and_sucrose_metabolism | 41 | 45 |
| Cysteine_and_methionine_metabolism | 42 | 43 |
| Porphyrin_and_chlorophyll_metabolism | 41 | 42 |
| Carbon_fixation_in_photosynthetic_organisms | 37 | 40 |
| Glycine-_serine_and_threonine_metabolism | 36 | 39 |
| Fatty_acid_metabolism | 38 | 38 |
| Glycerophospholipid_metabolism | 32 | 38 |
| Nucleotide_excision_repair | 34 | 37 |
| Glyoxylate_and_dicarboxylate_metabolism | 36 | 37 |
| Amino_sugar_and_nucleotide_sugar_metabolism | 36 | 37 |
| Phosphatidylinositol_signaling_system | 36 | 36 |
| Arginine_and_proline_metabolism | 34 | 36 |
| Photosynthesis | 35 | 35 |
| Proteasome | 34 | 34 |
| Citrate_cycle_-TCA_cycle- | 33 | 33 |
| DNA_replication | 28 | 33 |
| Glutathione_metabolism | 31 | 32 |
| Phagosome | 30 | 30 |
| 2-Oxocarboxylic_acid_metabolism | 30 | 30 |
| Protein_export | 29 | 29 |
| Alanine-aspartate_and_glutamate_metabolism | 27 | 27 |
| RNA_polymerase | 26 | 26 |
| Pentose_phosphate_pathway | 24 | 26 |
| Glycerolipid_metabolism | 23 | 25 |
| Inositol_phosphate_metabolism | 24 | 24 |
| Photosynthesis_-_antenna_proteins | 23 | 23 |
| Terpenoid_backbone_biosynthesis | 22 | 23 |
| Homologous_recombination | 15 | 22 |
| Fatty_acid_biosynthesis | 22 | 22 |
| N-Glycan_biosynthesis | 21 | 22 |
| Mismatch_repair | 18 | 22 |
| Phenylalanine-tyrosine_and_tryptophan_biosynthesis | 21 | 21 |
| Valine-leucine_and_isoleucine_degradation | 20 | 21 |
| Basal_transcription_factors | 21 | 21 |
| Arginine_biosynthesis | 21 | 21 |
| Sulfur_metabolism | 21 | 21 |
| Base_excision_repair | 16 | 20 |
| Pantothenate_and_CoA_biosynthesis | 19 | 20 |
| Propanoate_metabolism | 19 | 19 |
| Fructose_and_mannose_metabolism | 13 | 19 |
| Ubiquinone_and_other_terpenoid-quinone_biosynthesis | 18 | 19 |
| Nicotinate_and_nicotinamide_metabolism | 19 | 19 |
| Ascorbate_and_aldarate_metabolism | 18 | 18 |
| Sphingolipid_metabolism | 18 | 18 |
| Fatty_acid_degradation | 17 | 17 |
| Tyrosine_metabolism | 16 | 16 |
| ABC_transporters | 14 | 16 |
| Biosynthesis_of_unsaturated_fatty_acids | 16 | 16 |
| Nitrogen_metabolism | 10 | 16 |
| Selenocompound_metabolism | 14 | 16 |
| Galactose_metabolism | 14 | 16 |
| Carotenoid_biosynthesis | 14 | 15 |
| SNARE_interactions_in_vesicular_transport | 15 | 15 |
| Histidine_metabolism | 14 | 14 |
| One_carbon_pool_by_folate | 14 | 14 |
| Folate_biosynthesis | 11 | 13 |
| Regulation_of_autophagy | 12 | 13 |
| Biotin_metabolism | 12 | 12 |
| Arachidonic_acid_metabolism | 12 | 12 |
| Phenylalanine_metabolism | 12 | 12 |
| beta-Alanine_metabolism | 11 | 12 |
| Valine-_leucine_and_isoleucine_biosynthesis | 12 | 12 |
| Steroid_biosynthesis | 11 | 11 |
| alpha-Linolenic_acid_metabolism | 10 | 11 |
| Sulfur_relay_system | 11 | 11 |
| Pentose_and_glucuronate_interconversions | 11 | 11 |
| Glycosylphosphatidylinositol-GPI--anchor_biosynthesis | 10 | 11 |
| Plant_hormone_signal_transduction | 11 | 11 |
| Lysine_biosynthesis | 10 | 10 |
| Tryptophan_metabolism | 9 | 9 |
| Thiamine_metabolism | 9 | 9 |
| Monobactam_biosynthesis | 9 | 9 |
| Riboflavin_metabolism | 9 | 9 |
| Tropane-_piperidine_and_pyridine_alkaloid_biosynthesis | 8 | 8 |
| Butanoate_metabolism | 8 | 8 |
| Fatty_acid_elongation | 7 | 8 |
| Vitamin_B6_metabolism | 8 | 8 |
| Circadian_rhythm_-_plant | 8 | 8 |
| Isoquinoline_alkaloid_biosynthesis | 8 | 8 |
| Ether_lipid_metabolism | 6 | 7 |
| Non-homologous_end-joining | 5 | 7 |
| Cyanoamino_acid_metabolism | 4 | 5 |
| Other_glycan_degradation | 5 | 5 |
| Lysine_degradation | 5 | 5 |
| C5-Branched_dibasic_acid_metabolism | 5 | 5 |
| Linoleic_acid_metabolism | 4 | 4 |
| Lipoic_acid_metabolism | 3 | 4 |
| Taurine_and_hypotaurine_metabolism | 3 | 3 |
| Synthesis_and_degradation_of_ketone_bodies | 3 | 3 |
| Cutin-_suberine_and_wax_biosynthesis | 2 | 3 |
| Sesquiterpenoid_and_triterpenoid_biosynthesis | 2 | 2 |
| Caffeine_metabolism | 2 | 2 |
| Phosphonate_and_phosphinate_metabolism | 2 | 2 |
| Other_types_of_O-glycan_biosynthesis | 2 | 2 |
| Phenylpropanoid_biosynthesis | 1 | 2 |
|  |  |  |
